# Protocol of the baseline assessment for the Environments for Healthy Living (EHL) Wales cohort study

**DOI:** 10.1186/1471-2458-10-150

**Published:** 2010-03-23

**Authors:** Rebecca A Hill, Sinead Brophy, Huw Brunt, Mel Storey, Non E Thomas, Catherine A Thornton, Stephen Palmer, Frank Dunstan, Shantini Paranjothy, Roderick McClure, Sarah E Rodgers, Ronan A Lyons

**Affiliations:** 1 School of Medicine, Swansea University, Singleton Park, Swansea, SA2 8PP, UK; 2 National Public Health Service for Wales, Temple of Peace and Health, Cathays Park, Cardiff, UK CF10 3NW; 3 Centre for Child Research, Swansea University, Singleton Park, Swansea, SA2 8PP, UK; 4 Institute of Life Science, Swansea University, Singleton Park, Swansea, SA2 8PP, UK; 5 Department of Primary Care and Public Health, Cardiff University, Heath Park, Cardiff, CF14 4XN, UK; 6 Accident Research Centre, Monash University, Victoria, 3800, Australia

## Abstract

**Background:**

Health is a result of influences operating at multiple levels. For example, inadequate housing, poor educational attainment, and reduced access to health care are clustered together, and are all associated with reduced health. Policies which try to change individual people's behaviour have limited effect when people have little control over their environment. However, structural environmental change and an understanding of the way that influences interact with each other, has the potential to facilitate healthy choices irrespective of personal resources. The aim of Environments for Healthy Living (EHL) is to investigate the impact of gestational and postnatal environments on health, and to examine where structural change can be brought about to optimise health outcomes. The baseline assessment will focus on birth outcomes and maternal and infant health.

**Methods/Design:**

EHL is a longitudinal birth cohort study. We aim to recruit 1000 pregnant women in the period April 2010 to March 2013. We will examine the impact of the gestational environment (maternal health) and the postnatal environment (housing and neighbourhood conditions) on subsequent health outcomes for the infants born to these women. Data collection will commence during the participants' pregnancy, from approximately 20 weeks gestation. Participants will complete a questionnaire, undergo anthropometric measurements, wear an accelerometer, compile a food diary, and have environmental measures taken within their home. They will also be asked to consent to having a sample of umbilical cord blood taken following delivery of their baby. These data will be complemented by routinely collected electronic data such as health records from GP surgeries, hospital admissions, and child health and development records. Thereafter, participants will be visited annually for follow-up of subsequent exposures and child health outcomes.

**Discussion:**

The baseline assessment of EHL will provide information concerning the impact of gestational and postnatal environments on birth outcomes and maternal and infant health. The findings can be used to inform the development of complex interventions targeted at structural, environmental factors, intended to reduce ill-health. Long-term follow-up of the cohort will focus on relationships between environmental exposures and the later development of adverse health outcomes, including obesity and diabetes.

## Background

Health is the result of many interacting factors [1-3]. Foetal life and early infancy are important periods of susceptibility to environmental hazards, and exposures during early development can predispose the individual to disease such as diabetes, allergy, and asthma [4-8]. The foetus is susceptible to both historic and gestational maternal exposures via the placenta [8], such as maternal smoking and alcohol consumption. Adverse gestational environments have been associated with a number of sub-optimal birth outcomes, including decrements of foetal growth, early neurodevelopment, and respiratory health [9]. These health outcomes are postulated to result from 'foetal programming', whereby a stimulus or exposure at a critical, sensitive period of early life leads to permanent effects on individual structure, physiology, and metabolism [10]. Environmental exposures are equally significant in the early postnatal period. Because of the immature function of many of the organs, young infants have reduced elimination capacities, which means that they are less able than older individuals to excrete toxicants [11], making them more vulnerable to environmental insults.

Exposures during both the gestational and early postnatal period are identified as potential causes of chronic disease and other disorders in later childhood and in adulthood [12-15], or as potentially affecting birth outcomes [16]. However, while a growing number of studies are linking adverse health outcomes to a combination of gestational and postnatal environmental exposures, their focus tends to be on one type of exposure only, for example from passive smoking [17,18] or lead [19,20]. The tendency for many environmental epidemiological investigations to focus on single source characteristics, and the scarcity of data on exposure levels of multiple sources, is noted elsewhere [21]. The current evidence base therefore lacks a clear understanding of how multiple different types of exposures might act in concert to influence infant health.

Recommendations from studies which identify individual-level risk factors isolated from the environment in which they are situated can only be used by people who have the personal, social and economic resources to control their environment in such a way as to enable them to adopt the desirable health behaviours. This could widen social inequalities in health outcomes, with greater uptake of protective health behaviours (such as smoking cessation) observed in higher socioeconomic groups [22]. Evidence of this is borne out in individually targeted health promotion efforts which, while demonstrating efficacy in randomised trials, have limited population reach, especially with regard to disadvantaged groups [23]. Improvements in the health of populations (and reductions in the health differential within populations) can only occur when social and environmental structures facilitate healthy choices and behaviours in all sub-populations irrespective of personal resources.

## Methods/design

### Aim

EHL aims to take a multi-level analytical approach to the interaction of housing, neighbourhood, and parental/intrauterine environments with health outcomes, with a focus on both pre- and postnatal environments. Multiple health outcomes will be investigated, including but not limited to: intrauterine/postnatal growth and development, wheeze/asthma, allergies, diabetes, obesity, and unintentional injuries.

### Study design

EHL is a prospective, prenatally-recruited birth cohort study. It is designed to complement other epidemiology cohort studies around the world, e.g. [24,25], in order to validate and support their findings, and to increase statistical power whilst allowing for variation in context and exposure. In terms of its design, EHL has many similarities with other prenatally-recruited birth cohort studies, including the Avon Longitudinal Study of Parents and Children (ALSPAC), the Generation R study, the Danish National Birth Cohort study, and the Norwegian Mother and Child Cohort Study (MoBa) [26-29]. These similarities also extend to its collection of umbilical cord blood, which will provide an important resource for immediate and future epidemiological studies. However, due to issues of feasibility, we will not collect biological samples from mothers during pregnancy or DNA from the offspring, as has been done elsewhere (e.g. [30]). Finally, and in agreement with other birth cohort studies (e.g. [26,27]), we plan to develop a randomly-selected 'focus cohort' with which we can pursue more time-consuming data collection methods, including parental interviews and other air quality sampling techniques such as dust sampling. Related to this, formal collaboration with other birth cohort studies is currently a high priority.

Despite its similarities in design, EHL also departs from other birth cohort studies in some important respects. Our study focuses on research gaps which were identified at the two-day workshop held at the Wellcome Trust in 2008 to improve the research potential of existing longitudinal data. These gaps include objective, measured data as opposed to subjective data (e.g. accelerometer records of physical activity as opposed to questionnaire assessment), and measurements in pregnancy as opposed to postnatal baseline measures. Related to this, a major strength of this study is its use of home visits with study participants. These visits occur during the participant's pregnancy and enable objective measurements to be taken of the housing, neighbourhood, and parental/intrauterine environments. In this way, exposure information is collected by the researcher(s) in person, rather than by more remote methods such as telephone interviews as has been done in the Danish National Birth Cohort study [28]. As all home visits will occur during the gestational period, no mothers will be recruited to the study after the birth of their baby, which contrasts with the Generation R study [27]. Further, participants will not be requested to attend dedicated research centres for the purposes of the study, as they are elsewhere [27], thereby reducing the participant burden which may bias recruitment in birth cohort studies and risk substantial loss to follow-up [31]. In addition, this study responds to a recent call for new birth cohorts to involve fathers as a traditionally hard-to-reach, low-responding group in order to provide a fuller understanding of life-course epidemiology and the intergenerational transfer of health risk [32].

The focus will be on doctor-diagnoses of health outcomes (which will be uploaded and anonymously linked at the individual level in the SAIL databank) rather than parental report. Doctor diagnoses were selected as the optimum measure of outcomes because, and despite other findings to the contrary (e.g. [33,34]), parental report of symptoms has been shown to be sometimes inaccurate [35,36] and influenced by symptom understanding, perception, and underestimation [37-39], and the duration of recall and the seriousness of the health event being recalled [40]. This potential inaccuracy of parental report may in turn limit researchers' investigations of socio-demographic and ethnic variations in prevalence [37], and so will be avoided in this study.

Finally, this study is unique in collecting and linking electronically-held, person-based, routinely collected data for all participants. We have developed the Secure Anonymised Information Linkage (SAIL) databank, which links together a wide range of anonymised, person-based data from health and social care datasets, whilst complying with the requirements of data protection legislation and confidentiality guidelines [41]. This databank will enable us to conduct remote longitudinal follow-up of participants, thereby minimising sample attrition.

### Recruitment

Eligible participants will be pregnant women who: are aged 16 years or older, are resident in Wales, and are receiving their antenatal care within the Abertawe Bro Morgannwg (ABM) University NHS Trust. The ABM maternity service covers a large catchment area which includes women resident in Swansea, and parts of Neath Port Talbot, Powys, and Carmarthenshire. Women are provided with a study leaflet when they attend hospital for routine ultrasound scans or phlebotomoy. In addition, researchers will conduct face-to-face recruitment of women attending for routine ultrasound scans and/or blood tests in ABM maternity hospitals, and community midwives will provide study information to expectant mothers when they attend for antenatal appointments at GP practices. All researchers involved in recruitment will be familiar with a 'recruitment protocol', designed in order to give recruiters consistent and thorough information related to the study. Community midwives will only be expected to provide a brief overview of the aims of the study and a study leaflet which will contain the contact details of researchers involved in the study.

EHL is currently in its pilot phase to determine the efficiency of its recruitment and data collection processes. To date, and after the first five months of recruitment efforts, 137 women have expressed their interest to participate in EHL. Home visits are already underway with a good geographic spread and cross-section of the Swansea community in terms of household income, employment and housing conditions. No results from this pilot study are currently available.

### Sample size and geography

1000 families (mothers and babies) will be recruited to the study over the period April 2010 to March 2013. This sample size is comparable to the Environments for Healthy Living study conducted in Queensland, Australia [24]. Having the sample concentrated in one geographical area (in and around Swansea) is anticipated to assist the face-to-face recruitment of participants (either by researchers or local midwives) and the collection and storage of umbilical cord blood samples.

Swansea is located in south Wales, and is Wales' second largest city. Latest figures show that Swansea has a population of 229,100 and an annual birth rate of 2,700 [42]. Swansea's population is growing year-on-year, largely driven by inward migration from outside the UK [42,43]. Swansea has a mixture of different social backgrounds, housing types, and urban and rural areas. Currently, Swansea has the third largest black and ethnic minority population in Wales [44], and is ranked fifth highest amongst local authority areas in Wales for child poverty (the proportion of children in household with income poverty) [45]. The latest Welsh Index of Multiple Deprivation (2008) shows that Swansea has the single most income deprived (Castle 2) and single least income deprived (Killay North) areas in Wales [46]. Similarly, in terms of employment, Swansea has the second most deprived (Castle 2) and the least deprived (Killay North) areas in Wales [47]. This diversity of the population of Swansea will enable researchers to investigate area and locality effects and the spatial distribution of services, amenities and greenspace, and to conduct geographical mapping of incidence.

In terms of its size, EHL is smaller than other birth cohort studies. However, the EHL cohort recruited within Swansea will be linked and embedded within the longitudinal, total-population Wales Electronic Cohort for Children (WECC) of 35,000 births per year in Wales. WECC is an entirely electronic, anonymised child health prospective and retrospective cohort covering the entire Wales population, and is based upon routine electronic records. This type of e-cohort will be able to answer questions where exposures, outcomes and potential confounders are routinely collected or available through individual or ecological linkages.

### Data Collection Techniques

After providing their consent to be involved in the study, participants will be contacted by researchers in order to arrange a convenient date and time for a home visit. The home visit will occur at any time during pregnancy. At each home visit, consent will be obtained to collect six main sources of data (see Table 1):

**Table 1:** Data collection schedule

Gestational Environment	Data
Questionnaire	Income, employment, education, ethnicity, parity, home ownership, perceptions of home and neighbourhood quality, family dynamics, use of cleaning products in the home, smoking and alcohol consumption during pregnancy, supplements and medications taken during pregnancy, and plans and expectations for birth and feeding of baby

Anthropometric measurements	Parental height, weight, skinfolds and mid-arm circumference

Accelerometer	Physical activity of mother during pregnancy

Food diary	Parental dietary habits during pregnancy, including consumption of fats, sugar and salt (compared with Recommended Daily Amount [RDA] guidelines)

Umbilical cord blood	Immunological status of infant and antibodies from the mother

**Postnatal Environment**	**Data**

Home assessment	Hygiene, presence and extent of mould and/or damp, household hazards, safety features

Neighbourhood assessment	Air quality, accessibility of local facilities, availability of green space, walkability, proximity to major sources of noise, perceptions of local safety and crime

Noise meters	Noise levels in the home (parental bedroom)

Data Loggers	Temperature and humidity (parental bedroom and living room)

#### 1. Baseline questionnaire and anthropometric data

The questionnaire will be delivered by a touch-screen laptop computer in the participant's home during the home visit. Questions will include demographics of parents, information about relationships in the family, socio-economics, neighbourhood characteristics, and health, diet and lifestyle. In addition, an experienced anthropometrist will collect measures of upper-arm circumference; skinfold thickness (using Harpenden skinfold calipers) at the subscapular, suprailiac, tricep, and bicep; height (using a portable stadiometer); and weight (using Seca Scales). Anthropometric measurements will be taken from the mother at all times, and from the father when he is available and consenting.

#### 2. Diet and physical activity

Mothers and fathers will be requested to complete a seven-day diet diary, noting all meals (breakfast, lunch, dinner), snacks and drinks consumed each day. This will be recorded using a validated, self-reported seven-day food diary, supplemented by a questionnaire, for dietary assessment [48]. The food diary will be analysed by Health Options Ltd (Health Options Ltd, Cirencester, Gloucester, UK). Average daily kilojoules, percentage total fat, saturated fat, carbohydrate, protein and fibre will be calculated. Objective measurements of maternal physical activity will be achieved using Actigraph accelerometers [49]. Mothers will be requested to wear the accelerometer around the waist for seven continuous days, removing the device only for sleeping, bathing and swimming. The accelerometer will record data relating to the frequency and duration of light, moderate, and vigorous physical activity.

#### 3. Internal environment within the home

Measurements will be taken of noise levels, temperature, relative humidity, and nitrogen dioxide over seven days, beginning at the time of the home visit. A particular focus will be on the parental bedroom, as this is likely to be the place where the baby will sleep and spend several hours each day for at least the first few months of life. Noise levels will be measured in the parental bedroom, using a Bruel and Kjaer 2250 noise meter [50], to enable comparison with the World Health Organization's night noise guidelines for Europe [16]. Temperature and relative humidity will be recorded using Tinytalk II Data Loggers [51], in the parental bedroom and in another room in the home, one with mould or water damage if possible. Nitrogen dioxide will be measured using Palmes-type, passive diffusion tubes [52]. Four will be employed at each home visit: in the parental bedroom, the living room, the kitchen, and immediately outside the home in the vicinity of the front door, away from gas appliances on all occasions. The noise meter, Data Loggers, and diffusion tubes (as well as the diet diaries and accelerometer) will be collected by a researcher seven days after the home visit. In addition, a Home Assessment will be completed by the researcher with assistance from the participant/occupant. Information included in the Home Assessment will cover: the age of the home, and the presence and extent of double glazing, loft and cavity wall insulation, and mould and/or water damage. Observable mould and damp will be scored using a visual inspection of the walls. It was decided that total culturable fungi levels will not be collected (reflecting spores in the air) as more than nine repeated measures are needed for assessment of long term exposures [53]. A further aspect of the Home Assessment is the researcher's visual inspection of the home for hygiene, cleanliness and general presentation. The 'family cleanliness scale', used by social workers in the UK to assess children at risk of neglect, is used for this purpose [54]. Information will be recorded by the researcher(s) immediately after the home visit.

#### 4. External environment around the home

We are developing a system to link anonymised data at household levels to individual data across Wales, which involves the creation of Residential Anonymised Linking Fields (RALFs) [55]. A two-stage approach to measuring metrics from every household by the Health Information Research Unit (HIRU), and anonymisation of households by the NHS information organisation Health Solutions Wales (HSW), will allow environmental and health data to be combined from multiple sources without breaching confidentiality. Using the anonymised data, HIRU will use RALFs and accessibility metrics obtained from the RALF system to local amenities such as play facilities, shops, greenspace, and connectivity and the area walkability. In addition, noise data from the residences will be compared with locally available 'noise mapping' data in the given neighbourhood, which indicate proximity to three main noise sources, namely roads, rail and industry [56].

#### 5. Routinely collected (anonymised) NHS health data

Longitudinal follow-up of study participants and their children will be through routinely collected, anonymised data. Participants will be asked to consent to researchers having access to their maternity notes following the birth of their baby. These notes will provide data regarding the pregnancy (and previous pregnancies), family health histories, and the health and development of the baby up to age six weeks. Anonymised Linking Fields (ALFs) from the SAIL system will be used to link anonymised participant numbers with routine anonymised datasets from all GP records, hospital records (inpatients, out-patients, emergency departments), public health birth files, the National Community Child Health Database (NCCHD), the National Health Service Administrative Register, and foetal ultrasound scan results. Follow-up data on outcomes for health and unintentional injury in childhood will principally be obtained from the anonymised routine data. This includes data on body mass index, measured at the time of school entry, and available in the NCCHD dataset. In this way, we can link individual, household and neighbourhood data (from EHL) with health data (from SAIL). We will also be able to answer questions where exposures, outcomes, and potential confounders are routinely collected or available through individual or ecological linkages. Ecological linkages will be made when data are not available at an individual level but are at an area level.

#### 6. Umbilical cord blood

Cord blood will be collected by the midwife responsible for the mother and baby at the time of delivery within the ABM hospitals. Approximately 10-12 ml of cord blood will be collected in two labelled vacutainers (one heparinised and one for clotted sample), which will be couriered to Swansea University for processing and storage. Umbilical cord blood will be processed for preparation of (i) mononuclear cells for cryopreservation in liquid nitrogen, and (ii) plasma/serum that will be archived at -80°C. Other than the cord blood, no other biological samples will be collected.

After each home visit, study participants will be assigned an anonymised participant number. Thereafter, all data from the mother, father and infant will be linked to this number.

### Data Analysis

A multi-level analytical approach will be used to determine the various extrinsic and intrinsic influences on health and development. Multilevel regression models can be used to quantify the relationships between determinants (individual and group level variables) and outcomes, and in particular, the role of community-based interventions on outcomes in combination with multiple-level and individual risk factors. This approach can overcome common methodological barriers associated with conventional regression analysis in ecological epidemiology, where correlation among individuals sharing the same local environment is not accounted for. Multilevel modelling allows for the examination of variability in outcomes between individuals as well as between higher level units. It can also quantify the extent to which variability in outcomes are explained by variables defined at different levels.

### Analysis plan

Some areas of analysis and publications anticipated for the baseline assessment are [see 'Additional file 1']:

1. "Household build and respiratory health: a multilevel modelling approach"

2. "Projected cost effectiveness of structural interventions in the prevention of childhood asthma"

3. "Household cleanliness and impact on allergy and infection"

4. "Impact of exposures to pollutants on immune function at birth (using archived umbilical cord blood samples) and the development of allergies in childhood"

5. "Neighbourhood and noise: impacts on pregnancy and birth outcomes"

6. "Neighbourhood conditions and impact on obesity in pregnancy"

7. "Diet and activity in pregnancy and infant health at twelve months"

8. "The use of routine data to predict pregnancy complications and infant health"

9. "Household and neighbourhood characteristics and their impacts on unintentional injuries"

### Ethics

EHL has ethical approval from the South East Wales Research Ethics Committee (09/WSE02/37).

All data pertaining to the participants will be securely anonymised and encrypted so that individuals cannot be personally identified.

## Conclusion

EHL offers a unique methodology to understand how complex interactions between various levels of the epidemiological model contribute to overall health and wellbeing of populations. Data collection methods will be supplemented by routine data (see 'Additional file 2'). It is anticipated that EHL will contribute to current understanding of the associations between health and environmental conditions, and will also extend existing understanding by collecting information on multiple exposures.

The idea that the foetus is vulnerable to maternal exposures is a growing area of research, e.g. [10,12,14,15,57], to which EHL will contribute. It is linked with the research evidence that suggests that the characteristics of the gestational environment can 'program' developmental changes in the foetus, and that they persist after birth as postnatal traits [58] and into adulthood [59]. The research base for the contribution of postnatal environmental exposures to infant health is extensive, e.g. [60-62], but it lacks a clear understanding of how multiple exposures can affect susceptibility to ill-health and unintentional injury. EHL will investigate multiple gestational and postnatal exposures among a cohort of infants born to 1000 families in an area of South Wales covered by ABM NHS Trust.

Infant experience is argued to be an important determinant of later health due to the continued malleability of biological systems at this stage of development [63]. To this extent, addressing chronic illness in children and adults is likely to require interventions that reduce the adverse impact of social disadvantage in early childhood. While it is not possible to change all of the determinants of health, such as genetic predisposition, many environmental exposures are modifiable and preventable. Examples include the design of neighbourhoods and the quality of housing. Nevertheless, such exposures are often beyond an individual's control [64]. Understanding how to bring about structural, environmental change for the benefit of public health is an important challenge. It is particularly important given a growing body of evidence which points toward the limitations of individually targeted behavioural change interventions, in terms of population reach and lack of individual resources [23,65].

In response to calls to consider health within its social context [66], EHL will pay full attention to how health outcomes might be socially determined. It will examine how adverse social conditions, such as low income and living in deprived neighbourhoods, can have implications in terms of infant health. Its findings will contribute to a burgeoning literature on the life course approach in epidemiology, e.g. [67], by elucidating the impact of exposures during gestation and early post-partum on infant health. EHL will provide an evidence base to inform policy makers and practitioners of environmental exposures acting before birth and, after birth, inside and outside the home, and their impact on health outcomes. It will also assist these personnel in targeting publicly-funded environmental interventions to address public health in the future. We anticipate that the study findings will set a rationale for preventive interventions that focus on modifiable, structural factors in the built environment, and that demonstrate an appreciation of the social circumstances that can limit choice, thereby facilitating healthy behaviours and choices in all sub-populations.

One novel aspect of this study is the inclusion of geographical data to allow for spatial analysis of the effects of neighbourhood physical characteristics on lifestyle choices, physical activity, obesity, and injury prevention. The planned multi-level analysis is an innovative element that sets this study apart from the traditional risk-outcome analyses that characterise epidemiological cohort studies. Thus, a further contribution of EHL will be toward methodological developments in the discipline of epidemiology.

## Abbreviations

ABM: Abertawe Bro Morgannwg; ALF: Anonymous Linking Field; EHL: Environments for Healthy Living; HIRU: Health Informatics Research Unit; MRC: Medical Research Council; RALF: Residential Anonymous Linking Field; SAIL: Secure Anonymised Information Linkage; WECC: Wales Electronic Cohort for Children

## Competing interests

The authors declare that they have no competing interests.

## Authors' contributions

RAL conceived the idea of EHL. RH and SB drafted the manuscript. All authors read and approved the final manuscript.

## Pre-publication history

The pre-publication history for this paper can be accessed here:

http://www.biomedcentral.com/1471-2458/10/150/prepub

## Supplementary Material

Additional file 1 Additional file 1, **topic areas of investigation**. Anticipated areas of investigation (analysis plan) Click here for file

Additional file 2 **Data collection sources with links to routine data**. EHL data collection sources and routine data to measure the home and neighbourhood and gestational environments. Click here for file
