# Supplementary material for: Protocol of the baseline assessment for the Environments for Healthy Living (EHL) Wales cohort study
Source: BMC Public Health. 2010 Mar 23;10:150. doi: 10.1186/1471-2458-10-150 (PMC2850344; doi:10.1186/1471-2458-10-150)
Supplement: Additional file 1 — , topic areas of investigation. Anticipated areas of investigation (analysis plan) [file 1471-2458-10-150-S1.DOCX]

Additional file 1: Topic areas of investigation

| Theme | Topic Questions | Data | Impact |
| --- | --- | --- | --- |
| Home and neighbourhood environment | Air quality and respiratory illness – a multilevel approach | *Exposures and confounder:* Mould levels, socio-economic level (income education), housing type and age, family size, family history, household cleanliness, smoking in household.  *Outcome*: diagnosis (using GP records) of respiratory conditions (wheeze, asthma), hospital attendance for respiratory problems for members of the household (children, parents and other adults within the household). | Modelling of relative contribution of modifiable risk factors for respiratory illness. Estimates of cost effectiveness of structural intervention (insulation and ventilation, treating mould) and behavioural interventions (smoking, cleanliness). |
|  | Household cleanliness and allergy *vs.* infection | *Exposure and confounders*: Cleanliness (as assessed by researcher), cleanliness (as assessed by participant), pets, housing type, socio-economic level, family history of allergy.  *Outcome*: Attendance at the GP or hospital for diagnosis of allergy or infection. | Improvement in the evidence base to examine the Hygiene Hypothesis |
|  | Environmental determinants of pregnancy and birth outcomes | *Exposures and confounder:* Perceptions of the home, neighbourhood (Residential Environment Assessment Tool), family dynamics, expectations for the birth, noise levels, living conditions, walkability and connectivity of area, age of mother.  *Outcome:* Pregnancy and birth complications, gestation, mode of delivery, birth weight. | Modelling of relative contribution of structural and modifiable risk factors to pregnancy and birth outcomes (gestation, mode of delivery, etc.) and future health risks (delays in achieving milestones, etc.) |
| Gestational environment | Neighbourhood and impact on obesity in pregnancy | *Exposures and confounder:* Distance to recreational facilities (gym, greenspace), walkability of area, self reported safety, records of crimes in area, lifestyle factors (mother working, ownership of car, family size, socio-economic level, smoking, alcohol consumption, diet), co-morbidities, activity (accelerometer readings).  *Outcome*: Obesity during pregnancy (calculated from pre-pregnancy weight and weight gain during pregnancy and percentage body fat). | Estimation of the extent to which neighbourhood plays a role in obesity during pregnancy in order to inform the evidence base for neighbourhood design and new build planning. |
|  | Exposures in utero and the health of the infant | *Exposures and confounder:* Diet of mother, smoking and alcohol during pregnancy, exposures during pregnancy (analysis of cord blood for IgG, lead), cholesterol and other routine blood test results for mother.  *Outcome*: Health of infant, assessed by gestation, birth weight, complications at birth, health in first 6 months of life (GP records and attendance at hospital) | Modelling and extrapolation of the findings from the EHL to routine data on all births in Wales (e.g. the use of routine cholesterol levels as a predictor of diet and projected impact on birth weight and birth complications). |
