# Supplementary material for: Protocol of the baseline assessment for the Environments for Healthy Living (EHL) Wales cohort study
Source: BMC Public Health. 2010 Mar 23;10:150. doi: 10.1186/1471-2458-10-150 (PMC2850344; doi:10.1186/1471-2458-10-150)
Supplement: Additional file 2 — Data collection sources with links to routine data. EHL data collection sources and routine data to measure the home and neighbourhood and gestational environments. [file 1471-2458-10-150-S2.DOCX]

Figure 1: Data collection sources with links to routine data

Routine data:

Pollution maps

Noise maps

Home Assessment (in maternity notes)

Geographical mapping data (Residential Anonymous Linking Fields)

Routine data:

Maternity notes

Foetal ultrasound scans

EHL data collection:

Parental diet

Parental body composition

Maternal physical activity

Maternal medication use

Socio-economic status

Parental education

Family medical histories

Lifestyle/health behaviours

Umbilical cord blood

EHL data collection:

Noise

Temperature

Humidity

Nitrogen dioxide

Mould and damp

Home Assessment

Gestational environment

Home/neighbourhood
